# Supplementary figures and images for: Multi-omics analysis and single-cell sequencing revealed the lysosome associated molecular subtypes and prognostic model development of papillary thyroid carcinoma
Source: PLoS One. 2025 Jun 10;20(6):e0325486. doi: 10.1371/journal.pone.0325486 (PMC12151391; doi:10.1371/journal.pone.0325486)

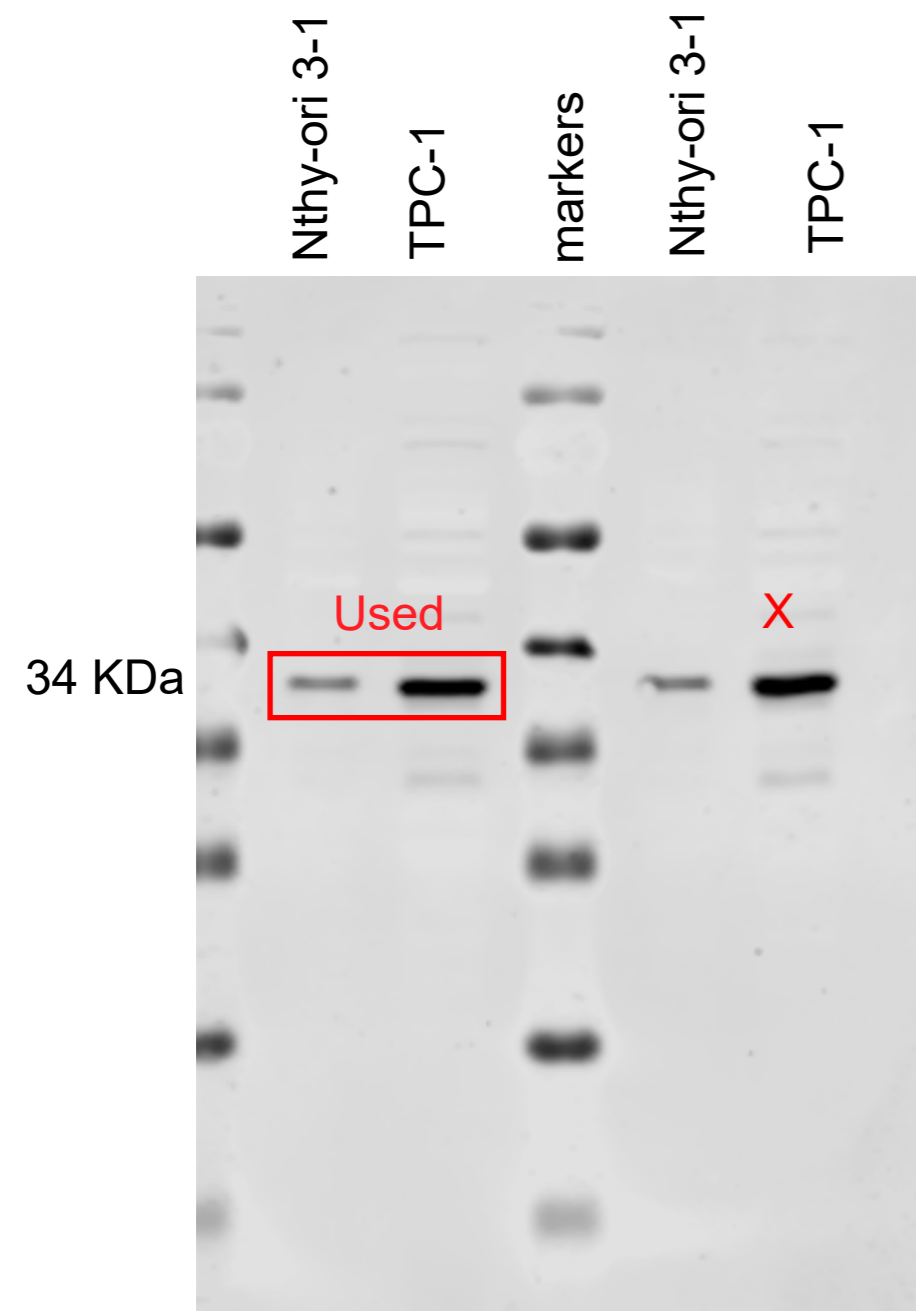

Fig 9A-DNASE2B

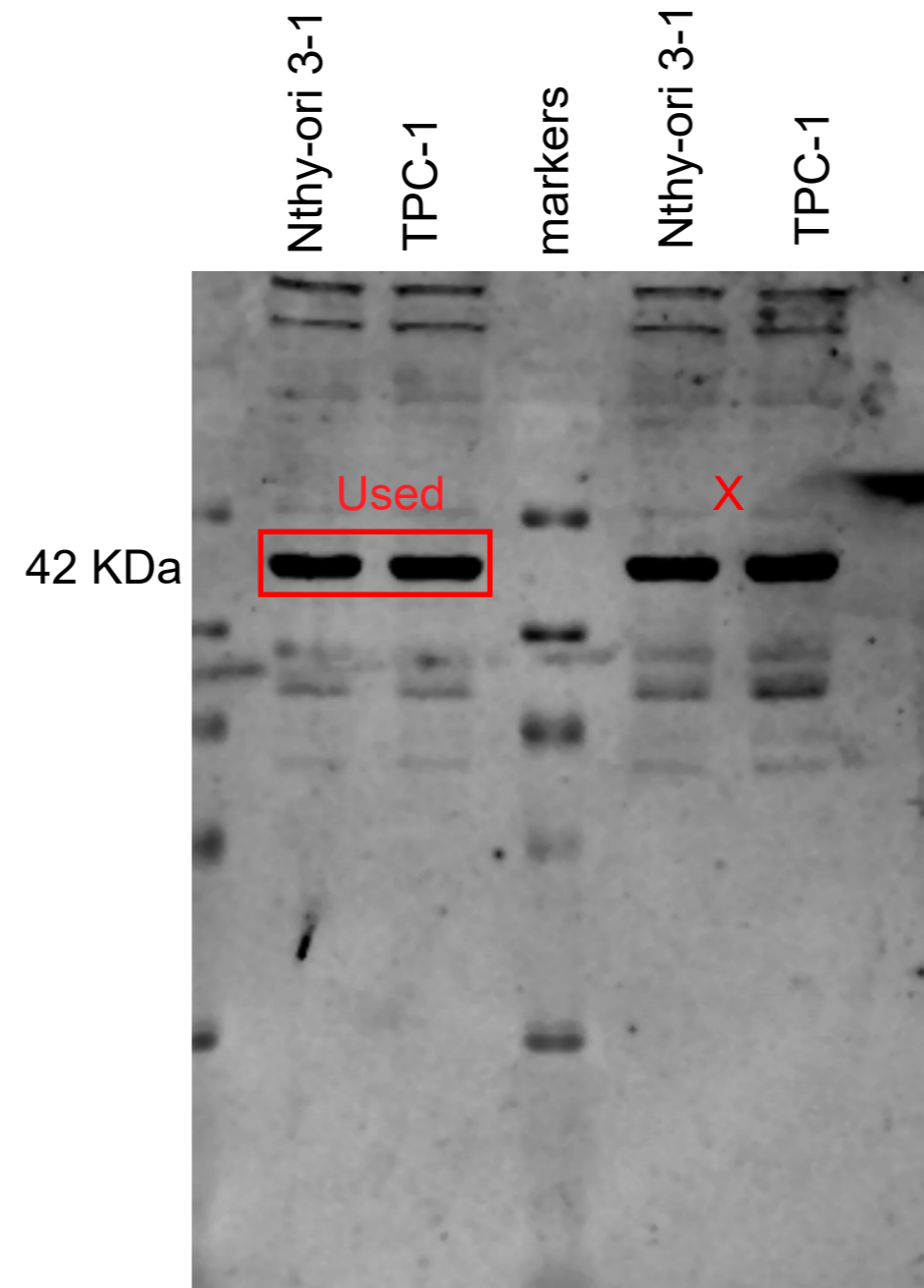

Fig 9A-actin

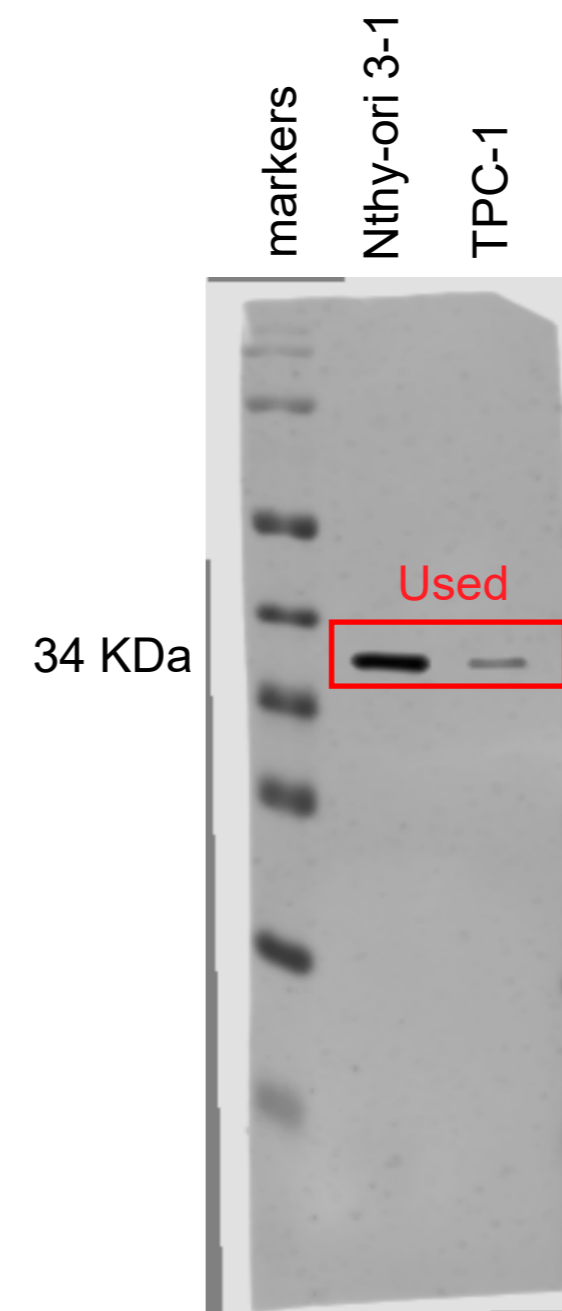

Fig 9C-DNASE2B

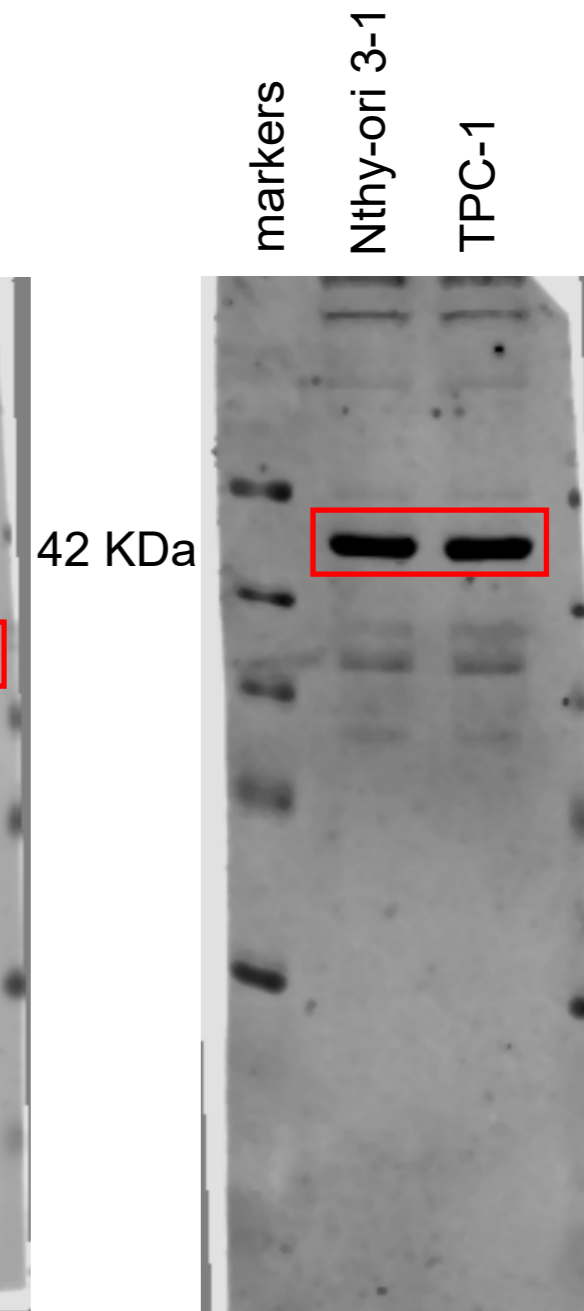

Fig 9C-actin

Supplement: S1 raw images — (PDF) [file pone.0325486.s004.pdf]
