# Supplementary material for: Multi-omics analysis and single-cell sequencing revealed the lysosome associated molecular subtypes and prognostic model development of papillary thyroid carcinoma
Source: PLoS One. 2025 Jun 10;20(6):e0325486. doi: 10.1371/journal.pone.0325486 (PMC12151391; doi:10.1371/journal.pone.0325486)
Supplement: Attachment — Submitted filename: Response to Reviewers.docx [file pone.0325486.s006.docx]

Dear Editor and Reviewers,

We sincerely appreciate your time and effort in reviewing our manuscript. Your insightful comments and valuable suggestions have been extremely helpful in improving the quality of our work. We have carefully addressed all the feedback and made the necessary revisions accordingly. Below, we provide detailed responses to each comment. We hope that our revisions meet your expectations, and we are grateful for any further suggestions you may have.

Reviewers' comments:

Reviewer #1: The Authors address the poorly studied topic of the role of lysosomes in the context of papillary thyroid cancer, which makes this work interesting. A weakness of the publication is the emphasis on the analysis of data generated by others. The amount of original data generated from the study is small and limited to simple in vitro assays on cell lines derived from human normal and cancer cells.

Below are suggested changes that should be made.

[1] It is worth mentioning that Nthy-ori 3-1 line is immortalized using the simian virus (SV)-40.

Reply：

Thank you for your question. In this study, we selected the Nthy-ori 3-1 cell line as the normal thyroid cell line based on the following considerations:

(1) Origin from Normal Thyroid Tissue: The Nthy-ori 3-1 cell line is derived from human thyroid cells and can accurately reflect the biological characteristics of normal thyroid cells. Therefore, it serves as an important tool for studying thyroid function and disease mechanisms.

(2) Immortalization: Through SV40-mediated immortalization, the Nthy-ori 3-1 cell line maintains the characteristics of thyroid cells while allowing sustained growth. This ensures stability and reproducibility in long-term experiments.

(3) Lack of Tumorigenic Transformation: Although this cell line is immortalized, it does not exhibit tumorigenic properties, making it suitable as a control group for comparative studies with thyroid cancer cell lines.

(4) Extensive Research Application: The Nthy-ori 3-1 cell line has been widely used in numerous studies, with a substantial body of experimental data and literature supporting its application. This well-established research background makes it an ideal model for normal thyroid cells.

Based on these advantages, we selected the Nthy-ori 3-1 cell line as the model for studying normal thyroid cells in our research.

[2] Please provide the exact details of the equipment used in the experiments (manufacturer and model name). This includes microscopes, chemiluminescence recording system, and microplate reader.

Reply：

Thank you for your review and valuable suggestions. We fully understand that detailed information about the experimental equipment is crucial for the reproducibility of research. Therefore, in the revised manuscript, we have supplemented the specific models and manufacturers of the equipment used. Once again, we sincerely appreciate your valuable feedback.

**Line 192-193, Line 212-213**

[3] Please provide (i) catalog numbers of all antibodies used in the Western blot analysis, (ii) the concentration of Tween 20 in TBS-T buffer, (iii) composition of buffer for dilution of primary and secondary antibodies.

Reply：

Thank you for your review and valuable suggestions. In the revised manuscript, we have incorporated this information. We believe that these additions will enhance the reproducibility of our study and provide other researchers with more detailed experimental insights.

**Line 202-204, line 206-210**

[4] Please complete the information in what 4% paraformaldehyde was diluted.

Reply：

Thank you for your review and valuable suggestions. In the revised manuscript, we have added this information. We believe that these additions will enhance the reproducibility of our study and provide other researchers with more detailed experimental insights.

**Line 236-238**

[5] Line 368: [5] Please add at the end of the sentence a reference to the Figures on which the described results on drug responses are presented, i.e., Figs. 7D-H.

Reply：

Thank you for your thorough review and valuable suggestions. In the revised manuscript, we have added the corresponding figure and table references to enhance the clarity and traceability of the results. Once again, we sincerely appreciate your careful review and constructive feedback.

**Line 400-404**

[6] Line 396: It is worth mentioning that papillary thyroid cancer is characterized by activation of the PI3K/AKT pathway (of which mTORC1 is a described component) and MAPK pathway, as described, for example, in Int J Mol Sci. 2021 Oct 31;22(21):11829 or Semin Cancer Biol. 2022 Feb;79:180-196.

Reply：

Thank you for your valuable suggestions. We fully agree with this perspective and have made the corresponding adjustments in the revised manuscript. Specifically, we have added references in line 396 to support the description of PI3K/AKT/mTORC1 and MAPK signaling pathway activation in papillary thyroid carcinoma (PTC). The following references have now been cited:

- *Int J Mol Sci.* 2021 Oct 31;22(21):11829
- *Semin Cancer Biol.* 2022 Feb;79:180-196

This revision strengthens the scientific rigor and credibility of our discussion. We sincerely appreciate your thorough review.

**Line 455-460**

[7] Please explain what the abbreviation “IPS” used on pages 11, 17, and 18 means.

Reply：

Thank you for your thorough review and valuable suggestions. IPS stands for Immunophenoscore, which is used to analyze the potential effects of immune-related therapies. We have added the full name of the IPS abbreviation in the revised manuscript to enhance the clarity and traceability of the results.

**Line 175**

Reviewer #2:

The authors have presented a study identifying lysosome-associated genes (LAGs) as potential prognostic markers in PTC. Some clarification and additional information as described below will strenghten the manuscript.

Reply：

Thank you for your suggestion. We validated the targets selected from the bioinformatics analysis through PCR, confirming their specific biological functions in thyroid cancer, thereby demonstrating the reliability of the bioinformatics results. We have rephrased this sentence in the revised manuscript to improve its readability.

**Line 71-72**

2：Line75- provide information about the 59 normal samples and 500 PTC, also clarify specific platform or technology use. (RNA-Seq, microarray)

Reply：

Thank you for your valuable feedback. We have added the clinical baseline data for the 59 normal samples and 500 PTC samples in the revised manuscript, along with details about the technical platform used. This addition enhances the transparency of the data description and provides readers with a clearer research background.

**Line 76-79**

3：Line78- Briefly explain why "limma" was chosen for differential expression analysis.

Reply：

Thank you for your review and valuable suggestions. In this study, we chose to use limma (package) for differential expression analysis, based on the following considerations:

1: Suitability for High-Throughput Data: limma was originally designed for microarray data but has been optimized for RNA-Seq data, particularly excelling in differential gene analysis when sample sizes are large but biological replicates are limited.

2: Robust Linear Model Approach: limma utilizes the empirical Bayes method, which allows for stable estimation of gene expression variability in small sample sizes, thus enhancing the reliability of statistical tests.

3: Wide Application and Validation: limma has been widely used in various transcriptomic data analyses, offering high computational efficiency and stability. It has been extensively applied in studies utilizing databases such as TCGA and GEO.

This addition strengthens the rationale for the selection of the analytical method.

**Line 88-93**

4：Line80- include what method was used to get adjusted p values

Reply：

Thank you for your valuable feedback. Regarding the issue in Line 80, we have added the method used to calculate the adjusted p-values in the manuscript. The p-value in the analysis was obtained using the limma package in R, which employs a linear modeling approach for differential expression analysis. Specifically, the p-value is derived from the statistical tests performed by the limma function, which calculates moderated t-statistics and p-values for each gene. These p-values are then adjusted for multiple testing using the Benjamini-Hochberg (BH) method to control the false discovery rate (FDR), resulting in the adjusted p-values (p.adjust) that were used to filter the significant genes in the analysis.

**Line 88-93**

5：Line85- Provide more details about LASSO implementation. Which type of LASSO was used? How was the penalty parameter chosen

Reply：

Thank you for your valuable suggestions regarding our study. Concerning Line 85 and the implementation details of LASSO regression, we have added a more detailed description in the revised manuscript.

In this study, LASSO (Least Absolute Shrinkage and Selection Operator) was implemented using the "glmnet" R package, which is widely used for fitting regularized linear models. LASSO regression uses L1 regularization, which penalizes the absolute values of the regression coefficients. This method promotes sparsity in the model by setting the coefficients of less relevant features to zero, thereby selecting a subset of significant variables (in this case, LAG-related genes) that contribute most to the model. The penalty parameter λ controls the strength of regularization. A higher λ increases the penalty, shrinking more coefficients to zero, while a lower λ allows for more variables to remain in the model. The optimal λ was selected using 10-fold cross-validation, a standard method in LASSO to find the value of λ that minimizes the cross-validation error.

We have included this additional information in the revised manuscript to provide readers with a clearer understanding of the specific details regarding the implementation of LASSO.

**Line 97-102**

6：Line 91- How was the "optimal survival cut-off value" determined?

Reply：

Thank you for your detailed review and valuable suggestions regarding our study. In response to the question about the "optimal survival cut-off value" in Line 91, we have added a more detailed explanation in the revised manuscript.

In this study, we used the "surv_cutpoint" function (from the survminer R package) to determine the optimal survival cut-off value. This method is based on the Maximally Selected Rank Statistics principle, which iteratively calculates different potential cut-off points and selects the one with the largest log-rank test statistic as the optimal cut-off. The specific calculation steps are as follows:

1: Calculate the log-rank statistic for each possible survival cut-off.

2: Select the cut-off point that yields the maximum log-rank statistic as the optimal cut-off.

3: Based on this cut-off, divide the samples into high-risk and low-risk groups.

We have included this detailed description of the analysis method in the revised manuscript to help readers better understand the process of selecting the optimal survival cut-off value.

Thank you again for your valuable feedback.

**Line 108-111**

7：Line97- Include citation for ConsensusClusterPlus and ssGSEA algorithm. Please make sure all the tools and databases used are properly cited.

Reply：

Thank you for your valuable suggestions. Regarding the issue you raised in Line 97 about referencing ConsensusClusterPlus and ssGSEA algorithms, we have made the revisions as per your advice. In the revised manuscript, we have added the appropriate literature citations for these two tools and algorithms to ensure that all the tools and databases used in our study are properly cited. Additionally, we have included supporting materials for databases such as the Cancer Immunome Database (TCIA), the Genomics of Drug Sensitivity in Cancer (GDSC), and Molecular Signatures Database (MSigDB).

Thank you again for your thorough review and suggestions. We greatly value your input, and we believe these revisions will enhance the quality of the manuscript.

8：Line253- LAG in TC?

Reply：

We sincerely apologize for any inconvenience caused by the writing errors in our manuscript. In the revised version, we have thoroughly reviewed the manuscript and corrected the mistakes. Thank you once again for your valuable feedback.

**Line 286**

9: Quantify the difference in survival outcomes between LAG subtype A and B and also include median survival times or hazard ratios with confidence interval

Reply：

Thank you for your valuable suggestion. To obtain the risk values and confidence intervals, Cox proportional hazards regression analysis is typically performed. Cox regression analysis provides the hazard ratio (HR) and its confidence interval for each factor, quantifying the impact of survival differences between groups. However, in this study, we used the log-rank test to examine the differences in clinical survival curves between different groups. The log-rank test is typically used to assess whether the survival differences between groups are statistically significant, but it does not directly provide risk values (hazard ratios) and confidence intervals. In the revised manuscript, we have updated the survival curve results to more clearly present the testing algorithm applied in our analysis. Thank you again for your feedback.

**Figure 2**

10: What parameters were used for GSVA analysis?

Reply：

Thank you for your valuable suggestion. We have included the parameters for Gene Set Variation Analysis (GSVA) in the revised manuscript. In this study, the specific parameters we used are as follows:

GSVA Analysis Parameters:

(1) Gene Set Database: We used the c2.cp.kegg.v7.2.symbols.gmt as the input gene set.

(2) Input Data: The gene expression data was preprocessed, including the removal of low-expression genes and normalization.

(3) Analysis Method: GSVA analysis was performed using the “GSVA” R package with the default Gaussian kernel to calculate the enrichment scores for each gene set in every sample.

(4) Parameter Settings: In the GSVA analysis, we set min.sz = 10 and max.sz = 500 to restrict the minimum and maximum number of genes in the gene sets, while selecting method = "gsva" and parallel.sz = 1 for score calculation.

We believe this addition will provide readers with more technical details about the GSVA analysis. Thank you again for your suggestion! If you have further questions or recommendations, we will continue to make adjustments and improvements.

**Line 120-123**

11: Line272 How was k=2 determined?

Reply：

Thank you very much for your comments. In this study, we primarily determined the molecular subtypes based on the following points:

(1) Consensus Matrix Plot: First, based on the consensus matrix plot, we found that the ambiguity level was lowest when K=2.

(2) Cumulative Distribution Function (CDF) Plot: The CDF plot is used to determine the most appropriate number of clusters. Generally, we observed the slope of the curves in the range from 2 to 9, and selected the cluster number corresponding to the curve with the gentlest slope (parallel to the x-axis). In this study, the red line represents the curve with the gentlest slope, so we chose to divide the samples into two groups.

(3) Delta Area: We used the Delta area for assessment. The Delta area curve compares the relative change in the area under the CDF curve between k and k-1. When k=2, we observed the smoothest change in the curve, thus we chose K=2 for subtype analysis and classification.

We believe these steps helped us accurately define the molecular subtypes.

12: Line410- Mention specific lysosome-related function

Reply：

Thank you for reviewing our manuscript and for your valuable comments. We have noted your suggestion regarding the need to explicitly mention specific lysosomal functions in line 410. We have revised this section accordingly. We have added information on the key role of lysosomes in cancer progression, particularly in regulating cell death, antigen presentation, and the immune microenvironment. Additionally, we have emphasized how lysosomal dysfunction may be associated with the onset and progression of PTC.

Thank you again for your suggestion. We believe this revision will further enhance the professionalism and completeness of the manuscript.

**Line 450-452**

13: It will be helpful to have some context in discussion section. Are there other studies that have investigated lys osmoses or prognostic markers in PTC? How the studies compare

Reply：

Thank you for reviewing our manuscript and for your valuable comments. We fully agree with your suggestion regarding the lack of sufficient background information in the discussion section. Research on lysosomes and related risk stratification in PTC is limited. To enrich the content, we will include relevant background information in the revised version, focusing on lysosomal functions and the study of prognostic biomarkers in tumor risk stratification. We have reviewed the relevant literature and will add several studies exploring the association between lysosomal dysfunction and clinical prognosis in cancer.

Thank you again for your feedback. Your suggestions will greatly help us enhance the quality of the manuscript.

**Line 512-521**

14: Some discussion of how LAG score could potentially be implemented in clinical practice can be useful.

Reply：

Thank you for reviewing our manuscript and for your valuable comments. We fully agree with your suggestion regarding the potential clinical application of the LAG score. In the revised version, we have elaborated on the role of bioinformatics analysis in tumor risk stratification, drug sensitivity analysis, and other aspects of personalized and precision medicine in the discussion section. Specifically, we will explore how the LAG score can assist doctors in assessing the immune microenvironment status of patients and how it can serve as an adjunctive diagnostic tool to help develop personalized treatment plans. Additionally, we will discuss how combining the LAG score with other conventional clinical markers can improve the accuracy and effectiveness of clinical decision-making.

Thank you for providing this important perspective for our research. We believe this will make our work more comprehensive and clinically valuable.

**Line 503-511**

Reviewer #3:

This is an interesting paper which I believe has a place in Plos One. It raises awareness of the importance of lysosome-associated genes (LAGs) as prognostic biomarkers of papillary thyroid carcinoma. There are however many points that the authors have to address before the paper is ready for publication.

1：Genes in the paper have to be in italics and not in regular font.

Reply：

Thank you for pointing out that gene names should be italicized rather than written in regular font. We have made the necessary revisions to the manuscript to ensure that all gene names are italicized. We appreciate your careful review, and we will continue to ensure consistency and accuracy in the formatting.

2：Line 273: “the results of the PCA plot showed a significant separation pattern of PTC”: Caution with using the word significant as it should only used when a p-value is obtained

Reply：

Thank you for your thorough review of our manuscript and your valuable feedback regarding the use of the word "significant." We fully understand your point and have made the necessary revisions in the revised version. Specifically, we have modified the statement in line 273 to avoid using the term "significant" unless supported by the corresponding p-value. The sentence has been revised to: "The results of the PCA plot showed a clear separation pattern of PTC," to more accurately describe the findings.

Thank you for your insightful suggestion, which has helped improve the scientific rigor of our paper.

**Line 305-307**

3：Line 291: “stromal score were significantly lower compared to LAG subtype A”: That’s not true because p-value is 0.068 and that’s larger than 0.05

Reply：

Thank you for reviewing our manuscript and for your valuable feedback regarding the statement in line 291. We have noted that the p-value of 0.068 is indeed greater than 0.05, and therefore the term "significantly" should not have been used. In the revised version, we have carefully proofread this section to ensure more accurate wording. Thank you again for your thoughtful feedback, which has helped us present our results more precisely.

**Line 326-329**

4：Line 298 “eosinophil infiltration was higher in LAG”: This is also wrong. It is very clear that the red boxplot indicating subtype B is higher than the blue boxplot indicating subtype A.

Reply：

Thank you for your thorough review of our manuscript and for pointing out the inaccuracy in the statement in line 298. We have carefully re-examined the data and confirmed the situation described by the reviewer, where the red boxplot (LAG subtype B) is indeed higher than the blue boxplot (LAG subtype A). As a result, we have corrected this statement in the revised version to ensure it is consistent with the actual data. Thank you for your valuable feedback, which has helped us improve the accuracy of our result presentation.

**Line 334-335**

5：Line 309 “LAG subtype B exhibited higher levels of LAG higher levels of LAG scores”: Add a line here saying that from this point onwards you will be referring to the clusters as high and low LAG clusters instead of Cluster A and Cluster B. Because later the colors of the boxplots in the figures are inverted.

Reply：

Thank you for your detailed review of our manuscript and for pointing out the need for further clarification regarding the naming of LAG subtypes in line 309. We understand the importance of this suggestion. In fact, in section 4 of the Results, we analyzed the role of the LAG scoring system in predicting the clinical prognosis of PTC. For Figure 4A, we examined the differential expression of the LAG scoring index across LAG molecular subtypes, so we described them as low and high LAG scoring subgroups. These actually represent different classification subgroups. To avoid confusion due to changes in the color order of the figures, we have revised the color scheme of the Sankey plot.

Thank you for your valuable suggestion. We believe this will help improve the readability and logical clarity of the paper.

6：Figure 4E, F: The ROC curves don’t look like any curves I have ever seen. Why are they so messy and look like an EKG. Also, why are there instances where the curves go higher than 1 in true positive rate? Please explain.

Reply：

Thank you for your detailed review of our manuscript and for raising concerns regarding the ROC curves in Figures 4E and 4F. We understand your concerns and have re-examined the process of generating the ROC curves.

First, regarding the issue of the curves being "overly erratic," we believe the possible reasons include: 1) the relatively small number of data points or potential overfitting in the classification model, which could cause significant fluctuations in the curve; 2) the ROC curves may not have been smoothed, leading to irregular shapes. We have rechecked the analysis process and optimized the generation of the ROC curves in the revised version, making them smoother and more intuitive. Secondly, regarding the issue of the True Positive Rate (TPR) exceeding 1 in certain areas, we have confirmed that this was due to a scaling issue during plotting, not an actual statistical calculation error. We have corrected this issue to ensure that all TPR values are strictly within the [0,1] range. In the revised version, we have provided the optimized ROC curves and included additional descriptions of the data processing methods in the figure legend to ensure that readers can correctly interpret the results.

Thank you again for your valuable feedback, which has helped enhance the scientific rigor of the study and the quality of data visualization. Should you have further suggestions, we would be more than happy to make adjustments and improvements.

**Figure 4E, F; Figure 5B, Line 355-357, Line 362-364**

7：Figure 5D: Please show the p-value associated with a X2 analysis that was done on the distribution of the groups. You can show the p-value in the figure next to each of the labels. Use different colors for stage because it is not easy to distinguish the different shades of green.

Reply：

Thank you for your detailed review of our manuscript and for your suggestions for improving Figure 5D.

（1）Addition of Chi-square (χ²) p-value: Following your recommendation, we have added the corresponding p-values in Figure 5D to clearly indicate the statistical differences between the groups. The p-values are now directly labeled next to the relevant data points, with different colors used to highlight significant differences, making it easier for readers to interpret.

（2）Optimization of the color scheme: We agree that the original color differentiation for the stages (stage) was not clear enough. Therefore, we have revised the color scheme and chosen more distinguishable colors to improve both the readability and visual effectiveness of the figure.

In the revised version, we have provided the updated Figure 5D and included additional descriptions of the statistical methods used in the figure legend. Thank you again for your valuable suggestions, which will enhance the quality and readability of the data visualization.

Figure 5D

8：Line 341: “CD56dim natural killer cells was notably upregulated in the LAG score subgroup B”: Here you say subgroup B but in the figure 6 it says high and low LAG scores...please be consistent

Reply：

Thank you very much for your review and for raising this question. We apologize for the confusion and any misunderstanding it may have caused.

In the second and third sections of the results, we primarily evaluated the molecular subtype characteristics of LAG in PTC, along with the immune therapy and immune microenvironment landscapes. Therefore, in Result 3, the findings in Figure 3 display the immune infiltration characteristics of the LAG molecular subtypes in PTC samples, along with the proportions of immune cell infiltration. Based on these results, we observed significant differences in the prognosis and immune infiltration landscapes of PTC samples between different LAG molecular subtypes.

Subsequently, based on the expression of these LAG markers and the clinical survival characteristics of PTC, we developed a novel LAG scoring index model. We then divided the PTC samples into two LAG score subgroups and evaluated the clinical survival outcomes and immune microenvironment features of these subgroups. Therefore, the results in Figure 6 mainly show the immune characteristics of the LAG score subgroups.

We appreciate you pointing out the error, and in the revised manuscript, we have reorganized the results in Figure 6 and adjusted the color scheme. Additionally, we have thoroughly reviewed the manuscript to ensure that all statements related to LAG grouping are consistent with the figures, to avoid confusion for the readers.

**Figure 6, Line 374-378**

9：Figure 6: In 6A high is blue, but in 6B, high is red. Please be consistent across the paper

Reply：

Thank you for your thorough review of our manuscript and for pointing out the inconsistency in naming. We fully agree with your observation that consistency is crucial for improving the readability of the paper. In the revised version, we have reorganized Figure 6 to avoid any confusion for the readers. We greatly appreciate your valuable suggestion, which has contributed to enhancing the logical flow and academic rigor of the manuscript.

**Figure 6A**

10：Line 358: LAG scoring subgroups were associated with biological functions such as long-chain fatty acid transport detoxification of copper ion, stress response to copper ion, high-density lipoprotein particle, and plasma lipoprotein particle”: What does that mean? Why is that exciting? How is it relevant to the disease of interest?

Reply：

Thank you for your thorough review of our manuscript and for raising the issue regarding the statement in line 358. We recognize that the current wording may be too general and fails to adequately explain the connection between these biological functions and the diseases under investigation, as well as their potential significance.

In the revised version, we have expanded the discussion on the potential roles of these biological functions in papillary thyroid carcinoma (PTC). For example, long-chain fatty acid transport plays a crucial role in the metabolic reprogramming of cancer cells and may influence the energy metabolism and proliferative capacity of PTC. Additionally, detoxification of copper ions and the stress response to copper ions may be involved in the regulation of oxidative stress within the tumor microenvironment, while lipoprotein particles have been reported to be associated with cancer cell signaling and inflammatory states.

Furthermore, we have further elaborated on how these functions might impact the progression of PTC, potentially relating to patient prognosis, treatment response, or tumor microenvironment regulation, thereby enhancing the clinical relevance of these findings. We believe this revision will contribute to the scientific robustness and clinical translational value of this section.

**Line 489-502**

11：Figure 8: The violin plots are showing cells that must have been removed. You said in the method section that cells with percent.mt > 5% will be removed… but looking at the figure we see a lot of cells higher than 5% mt content. In the method section you will need to elaborate more on the seurat object preparation. Did you remove doublets? Did you remove immune cells invading the tumor? What did you use to integrate samples? CCA or Harmony? You need to specify the resolution chosen when running findclusters() function.

Reply：

Thank you for your thorough review of our manuscript and for pointing out the potential inconsistency between Figure 8 and the methods section. We fully agree that ensuring the clarity and transparency of the data processing methods is crucial for the reproducibility of the research.

As you mentioned, in Figures 8A and 8B, we primarily present the reliability of the sequencing data from the seven PTC samples and the quality control (QC) metrics used to assess the data. As shown in Figure 8A, the violin plots reflect key metrics for these seven PTC samples:

1：The number of genes detected per cell (nFeature_RNA), where a low count may indicate empty or low-quality cells, and a high count may suggest doublets.

2：The total transcript count per cell (nCount_RNA), also known as the Unique Molecular Identifier (UMI) count, which represents the total number of transcripts in each cell. A high count may indicate doublets, while a low count could indicate low-quality cells.

3：The proportion of mitochondrial genes (percent.mt), which calculates the percentage of UMI from mitochondrial genes and is commonly used to assess cell vitality. A high percentage could indicate stressed or fragmented cells.

Through Figure 8A, we visually assess the quality of the single-cell data from these PTC samples and set reasonable filtering criteria to exclude low-quality cells and cells with high mitochondrial content (nFeature_RNA > 200 & nFeature_RNA < 2500, percent.mt < 5).

Based on this, we performed data filtering and normalization for each sample and identified the top 2000 highly variable genes for subsequent dimensionality reduction analysis (using the FindVariableFeatures function, selection.method = "vst"). We then employed the "harmony" algorithm to integrate the samples, removing batch effects and conducting dimensionality reduction. Additionally, we performed clustering using the findClusters function with a resolution setting of 1.2. Generally, higher resolution values lead to more cell clusters, and a resolution of 1.2 provided optimal results.

Once again, thank you for your valuable suggestions. In the revised manuscript, we have added detailed explanations to the methods section to make this part of the description clearer.

**Line 153-163, line 166-171**

12：Line 379: “A visualized heatmap illustrated the expression characteristics of marker genes in each cell subpopulation”: How many marker genes are there? Where did you get these genes from? Are they 2000 differentially expressed genes? Need to provide the list as supplementary material. Cell Annotation using SingleR: How did you annotate? Where did you get the reference data from? Or did you use your own marker genes that you obtained from reading the literature? Provide this list of markers in a supplementary table, please.

Reply：

We sincerely appreciate the reviewer’s in-depth analysis of our study. Your comments are very important, and we have made corresponding revisions to enhance the transparency and reproducibility of the research.

1：On the number and source of marker genes: In the heatmap, we display representative marker genes for each cell subgroup. These marker genes were identified using the FindAllMarkers function, employing the Wilcoxon rank-sum test, with filtering criteria of logFC > 0.25 and p-value < 0.05.

2：Not all 2000 differentially expressed genes (DEGs) were used for marker gene selection: In our manuscript, we selected the top 2000 genes primarily for dimensionality reduction analysis. For single-cell RNA sequencing, the goal of selecting the top 2000 highly variable genes is to extract biologically meaningful genes, remove low-variance and noisy genes, optimize dimensionality reduction and clustering, and enhance cell type differentiation ability, thus improving the stability and accuracy of the data analysis.

To increase data transparency, we have provided a complete list of all marker genes as supplementary material (Supplementary Table 3) in the revised manuscript.

3：On the SingleR cell annotation strategy: Cell annotation was performed using the SingleR package, which matches unknown cells to reference datasets of known cell types based on correlation. In this study, we used the Human Primary Cell Atlas (HPCA) as the reference dataset to ensure the accuracy of the annotations. The HPCA is one of the commonly used reference gene sets in the SingleR package, primarily for cell type annotation in single-cell data. This dataset, sourced from the Blueprint and FANTOM5 projects, includes gene expression profiles from different human tissues and cell types. Using the “celldex” R package, we were able to directly access the HPCA data without the need for separate downloads. In the revised manuscript, we have included the list of marker genes.

We are grateful for the reviewer’s valuable suggestions, and we believe these revisions will further enhance the readability and reproducibility of our study. Should there be any further comments, we would be happy to continue improving the manuscript!

**Line 153-163, line 166-171, Supplementary Table 3**

Reviewer #4:

Comment 1:

In Figure 1B, the authors present four protective factors (HR < 1). However, the hazard ratios of three out of four protective factors are very close to 1, suggesting a minimal effect or no meaningful impact. Additionally, regarding the clinical survival outcomes used in the univariate Cox analysis, do the authors employ overall survival (OS) or progression-free survival (PFS)?

Reply：

We sincerely thank you for your careful review of our manuscript and for your valuable comments.

1：Regarding the HR values of protective factors in Figure 1B being close to 1:
We appreciate the reviewer’s in-depth analysis of the results. In the first part of the results, we first analyzed the differential expression of the LAG signature in normal and PTC samples. Subsequently, using univariate Cox analysis, we assessed the prognostic value of these DE-LAG signatures in PTC. Based on this, we further used multivariate Cox analysis to identify variables with independent prognostic value and constructed the LAG score prognostic model, as shown in Figures 1D-E. Indeed, as pointed out, the HR values of the three protective factors in Figure 1B are close to 1, indicating that these factors may have minimal or no significant clinical impact. These factors may be related to other variables or background conditions, and while the HR values are close to 1, they may still exhibit a certain degree of protective effect under specific circumstances.

2：Regarding the clinical survival outcome used:
Regarding the clinical survival outcome mentioned in the univariate Cox analysis, we used overall survival (OS) as the primary analysis metric. We have clearly indicated this in the manuscript.

Once again, thank you for your careful review and valuable suggestions. These revisions will help improve the accuracy and completeness of the manuscript.

**Line 95**

Comment 2:

In Figure 2F, the authors report gene signatures enriched in LAG subtypes A and B. It would be valuable for the authors to discuss these findings in the context of potential biological mechanisms. Specifically, how do these gene signatures contribute to the observed survival differences between the two subtypes?

Reply：

We sincerely appreciate your valuable feedback. In response to your comments regarding the gene characteristics of LAG subtypes A and B in Figure 2F, we have conducted a thorough discussion and made revisions accordingly. We fully agree with your suggestion and have selected type 2 diabetes-related characteristics as factors influencing the clinical manifestations and survival differences of the different subtypes, and we have briefly discussed this aspect.

Thank you once again for raising this important issue, as it has allowed us to present a more comprehensive understanding of the potential biological significance of our findings.

**Line 317-321**

Comment 3:

In Figure 3, the authors compare immune cell infiltration between the two subtypes. While some p-values are statistically significant, the differences in immune infiltration and stromal scores appear quite subtle. Could the authors clarify whether these small differences could be attributed to noise? Additionally, what statistical method was used to compare these immune scores between the two subtypes?

Reply：

We sincerely thank you for your meticulous review of our manuscript and for your valuable suggestions. In response to your question regarding the differences in immune cell infiltration and stromal scores in Figure 3, we have made the corresponding revisions and additions to the manuscript. Below are our responses:

1: Explanation of immune cell infiltration and stromal score differences:
We understand your concern regarding the relatively small differences in immune infiltration and stromal scores. While some p-values are statistically significant, as you pointed out, these differences may indeed be subtle. However, we believe that these small differences are not entirely due to noise. They may reflect subtle changes in the immune microenvironment and stromal composition between different subtypes. Although these changes are small, they could have biological significance in specific clinical contexts. We also acknowledge that these subtle differences may arise from sample heterogeneity and data variability, but we have taken rigorous data cleaning and correction steps to minimize the impact of noise.

2: Statistical methods for immune score comparison:
For the comparison of immune cell infiltration and stromal scores, we used the Wilcoxon rank-sum test (for comparisons between two groups) and performed multiple validations to ensure the reliability of the statistical results. Additionally, we conducted sensitivity analysis to assess the impact of different statistical methods on the results. In the revised manuscript, we have provided a detailed description of the statistical methods used to ensure the transparency and reproducibility of the results.

Once again, we appreciate your valuable suggestions, which have helped us clarify the interpretation of the results and enhance the rigor of the manuscript.

Comment 4:

In Figures 4C and 4D, the authors state that they divided PTC samples into two independent cohort subgroups to validate the LAG scoring system's predictive ability for clinical outcomes. However, the LAG molecular subtypes and LAG scores were developed using the same TCGA PTC dataset. Even though the dataset was split for validation, it still involves reusing TCGA PTC samples. Could the authors validate these results using an entirely independent dataset?

Reply：

We sincerely appreciate your meticulous review of our manuscript and your very important suggestions. We fully agree with your point that including an external validation cohort could further confirm the reliability and predictive ability of the LAG score model we have developed.

In the GEO database, we searched for potential PTC datasets; however, unfortunately, most of the PTC datasets do not contain survival time and survival status, and the sample sizes are relatively small. Therefore, these datasets are not suitable for inclusion in our current data analysis. We are grateful for your valuable guidance on this matter.

In the current study, we are continuously enrolling clinical samples in our clinical research and conducting follow-up observations, with the aim of further validating the reliability and stability of the prognostic model we have constructed from a clinical perspective.

**Line 524-530**

Comment 5:

Similarly, for Figures 5B and 5C, it would be beneficial to validate the model using an independent dataset to confirm its predictive performance.

Reply：

We sincerely thank the reviewer for highlighting this critical issue. As you mentioned, validating the model with an independent dataset could further clarify its predictive performance. Unfortunately, in the GEO database, we found that the PTC datasets do not provide survival time and survival status for the samples. Therefore, we performed stability validation of the model using an internal random cohort division algorithm.

We appreciate you pointing out this limitation. In the revised manuscript, we have added a discussion on this limitation in the discussion section. In future research, we plan to conduct multi-center and large sample clinical cohort analyses to further validate the clinical applicability of this model.

**Line 524-530**

Comment 6:

In Figures 7D–H, the authors compare the predicted drug sensitivity between the two groups. However, the observed differences appear relatively small. Could the authors provide a positive control, such as tumor samples known to be sensitive to a specific drug, and compare them to resistant tumor types? This comparison would provide context for evaluating the magnitude of the observed differences. Additionally, in Figures 7B and 7C, it would be helpful if the authors could further interpret the KEGG enrichment analysis and explain how these pathways might relate to sensitivity to the predicted IC50 values of different drugs.

Reply：

We sincerely appreciate the valuable feedback provided by the reviewer. In response to the issues raised, we have conducted further analysis and made revisions to the manuscript.

(1) Regarding the small differences in drug sensitivity, we acknowledge the significance of this issue. In our analysis, we found that drug sensitivity prediction models have certain limitations. These models typically rely on gene expression data, drug target information, and other molecular features to estimate drug IC50 values. However, the results of these predictions may be limited by the quality of the available data and the accuracy of the prediction methods and algorithms. Predicted drug sensitivity may not fully reflect actual clinical drug responses, especially as discrepancies can arise between in vitro and in vivo responses. We appreciate your suggestion to compare tumor samples known to be sensitive to specific drugs with those that are resistant to provide context for evaluating the magnitude of observed differences. Unfortunately, we were unable to find data on PTC drug resistance in the GEO database. We have added this limitation in the discussion section of the revised manuscript.

(2) Regarding the KEGG enrichment analysis in Figures 7B and 7C and its relationship to drug sensitivity: We have further clarified the interpretation of the KEGG enrichment analysis in the revised manuscript. In the updated discussion section, we provide a more detailed analysis of the key pathways identified in relation to drug sensitivity, such as copper metabolism and lipid metabolism pathways, and their impact on drug resistance. Additionally, we summarize potential mechanisms through which lysosomes affect drug resistance, including physical binding, cell cycle regulation, and activation of signaling pathways, which ultimately influence the tumor's drug response.

Once again, we thank you for your invaluable suggestions, which have greatly improved the clarity and depth of our research. Should you have any further questions or additional suggestions, we would be happy to make further revisions.

Comment 7:

For Figure 8, the single-cell sequencing dataset includes seven PTC samples. It is unclear what novel insights can be gained from this single-cell RNA sequencing analysis. Since four out of five LAG genes are almost undetectable in this dataset, and DNASE2B is expressed in all cell types, the study does not appear to identify cell-type-specific enrichment. Could the authors clarify the biological significance of this analysis and whether any new findings emerge from it?

Reply：，

We sincerely appreciate the valuable feedback you provided on our research. In response to the issues raised, we have conducted further analysis and made revisions to the manuscript. Below are our responses:

1: On the new insights from single-cell RNA sequencing analysis: We understand your concerns regarding the single-cell RNA sequencing analysis. Indeed, in this study, we used single-cell RNA sequencing data from seven papillary thyroid carcinoma (PTC) samples. In our analysis, we focused on the expression patterns of LAG genes across different cell types. While some LAG genes exhibited lower expression levels, this observation provides valuable insight: these genes may not be universally active in the PTC tumor microenvironment but rather regulated by tumor-specific immune or other cellular pathways. This explains why the expression differences of these genes in various cell types are critical for understanding tumor immune evasion and the characteristics of the microenvironment.

2: On the low expression of LAG genes and the widespread expression of DNASE2B: You pointed out that most LAG genes were hardly detectable in the single-cell data, while DNASE2B showed notable expression across all cell types. In response, we have further clarified this issue in the manuscript. DNASE2B, as a widely expressed gene, likely reflects the biological characteristics of tumor cells in PTC, while the low expression of LAG genes suggests potential regulatory mechanisms or immune evasion mechanisms in PTC. Although we did not observe a clear enrichment of LAG genes in all cell types, we still analyzed the expression patterns of LAG genes and highlighted their potential immune role in the tumor microenvironment, providing direction for future research. We agree with your point that the results in Figure 8H did not clearly convey our findings. In the revised manuscript, we have reanalyzed this part and included a more detailed description of the LAG signature's expression characteristics in different cell types, further elucidating the potential role of LAG genes at the single-cell level in PTC.

Overall, while the single-cell RNA sequencing analysis in our study did not yield significant new findings regarding cell type-specific enrichment, it has provided valuable clues for a deeper understanding of immune evasion mechanisms in the PTC tumor microenvironment.

Once again, we thank you for your constructive suggestions, and we believe these revisions will enhance the depth and clarity of the research.

**Figure 8H, Line 422-428, line 818-819**
