# Supplementary material for: Multi-omics analysis and single-cell sequencing revealed the lysosome associated molecular subtypes and prognostic model development of papillary thyroid carcinoma
Source: PLoS One. 2025 Jun 10;20(6):e0325486. doi: 10.1371/journal.pone.0325486 (PMC12151391; doi:10.1371/journal.pone.0325486)
Supplement: Attachment — Submitted filename: Response_to_Reviewers_auresp_2.docx [file pone.0325486.s007.docx]

Dear Editor and Reviewers,

We sincerely appreciate your time and effort in reviewing our manuscript. Your insightful comments and valuable suggestions have been extremely helpful in improving the quality of our work. We have carefully addressed all the feedback and made the necessary revisions accordingly. Below, we provide detailed responses to each comment. We hope that our revisions meet your expectations, and we are grateful for any further suggestions you may have.

Reviewers' comments:

Reviewer #3: I thank the authors for submitting a review of their manuscript in which they address most of my points. Most points have been addressed to my satisfaction. There are two points that the authors need to address for the paper to be ready for publication.
The authors did a good job changing the color scheme for figure 5D, however in doing that they chose a color scheme for “gender” that is hard to look at: Red/Brown. The colors they had in the first submission were better: Red/Blue.

Reply:

Thank you very much for your helpful suggestion regarding the color scheme used in Figure 5D. We appreciate your comment that the red/brown scheme we adopted in the revision was difficult to interpret. Following your recommendation, we have changed the color scheme for the “Gender” variable back to Red/Blue, as it provides clearer visual distinction. The updated figure has been included in the revised manuscript, and we have ensured consistency in color usage across all relevant figures.

Thank you again for your thoughtful feedback and support.

**Figure 5D**

With regards to the single cell portion, they made improvements to the writing and explained the methods clearly, however I am concerned with their interpretation. PTC is a solid tumor and would therefore have clearly defined neoplastic cells. Looking at the figures 8F and 8G, we do not see tumor cells. Instead, we see a lot of immune cells and in addition to smooth muscle, epithelial, and endothelial. Is it normal to find smooth muscle in a PTC tumor? I do not think so. What is happening is that they are using the cells present in the reference: celldex and HPCA, which might not have the right reference for the cells that are truly present in PTC. It makes no sense that the majority of cells are immune cells, Where are the tumor cells? The algorithm is forced to label the cells and I believe it is labeling them with immune labels just because the reference is not optimized for PTC. The only way to fix this is to sit down with a pathologist and discuss gene markers for cell types that usually are present in PTC. Also, you will need to run gene ontology on the DEGs of the cell clusters that you have and see if you can guess what type of cells they are based on ontology. Also, have a conversation with a single cell transcriptomics expert to contribute to your data analysis and interpretation.
Good job on what you have done so far. You are near the finish line!
Best wishes,

We sincerely appreciate the reviewer’s professional and insightful comments. Your questions are highly pertinent and constructive. After discussion with the senior members of our research group, we would like to provide the following responses:

**1. Why were most of the cells annotated as immune cells rather than tumor cells?**
This is indeed a critical question and reflects a common technical challenge in single-cell RNA sequencing (scRNA-seq) analysis of solid tumors, especially in complex and heterogeneous tumors such as papillary thyroid carcinoma (PTC).
(1) First, there is no standard annotation label for “tumor cells” in scRNA-seq datasets. Most commonly used annotation tools (e.g., SingleR, Seurat, ScType) are designed to identify normal cell types, such as epithelial cells, dendritic cells, macrophages, and T cells. These tools typically do not explicitly label cells as “tumor cells,” even when their gene expression profiles suggest tumor-like features.
(2) The transcriptomic profiles of thyroid tumor cells are often highly similar to those of normal thyroid epithelial cells. In PTC, tumor cells may still express thyroid-specific markers such as *TG*, *TPO*, *PAX8*, and *TTF1*. As a result, many tumor cells may be annotated as epithelial or other cell types. Furthermore, the transcriptomic resemblance between PTC tumor cells and normal epithelial cells poses a challenge for automatic annotation. To clearly distinguish between normal and malignant epithelial cells, additional analyses such as CNV inference or mutation integration are required. This cannot be achieved solely through transcriptomic data.

**2. Is the presence of smooth muscle cells in PTC tumors normal?**
Indeed, this is an important yet often overlooked issue. It is possible, though uncommon, to find smooth muscle cells associated with vasculature or stromal components within PTC tumor tissues. However, their proportion is usually low. In our single-cell analysis, we employed general reference databases such as *celldex* and *HPCA*, which are trained on gene expression profiles from peripheral tissues and not specifically optimized for thyroid or tumor tissues. This may have led to some fibroblast-like or stromal cells being misannotated as smooth muscle cells. Under the guidance of experts in single-cell analysis, we re-examined the expression of smooth muscle markers (*ACTA2*, *TAGLN*, *MYH11*) and found that this annotation was inaccurate. These cells are more accurately classified as myofibroblasts rather than smooth muscle cells. We have revised this annotation in the revised manuscript, and we are grateful for your question, which helped us further refine our results.

**3. Was it inappropriate to use celldex and HPCA as reference datasets for PTC?**
This is indeed a critical issue that warrants attention. Currently, there is no standardized reference database specifically designed for thyroid cancer, particularly PTC. However, the Human Primary Cell Atlas dataset project includes single-cell data from normal thyroid tissue, encompassing epithelial cells, follicular cells, and immune cells. Because of its higher tissue specificity, we used this database for annotation and performed manual correction with the aid of the CellMarker database. Your comment highlights the importance of constructing a PTC-specific reference gene set to enable more precise cell-type annotation, which will be a focus of our future work.

**4. On the source of the data**
It is important to note that the origin of the samples plays a vital role in data interpretation. The scRNA-seq datasets used in our study were obtained from public databases (PMID: 34663816; PMID: 36523593; PMID: 38609408). Upon careful review of these original studies, we noted that tumor cells were also not explicitly annotated in their results. In contrast, our annotations appear to be more detailed than theirs, possibly due to differences in analytical strategies (the above datasets were sequenced and published in 2021).

**5. GO analysis of DEGs to aid in cell-type inference**
We performed GO enrichment analysis on the differentially expressed genes (DEGs) for each cell cluster. The results showed significant enrichment of immune-related pathways, such as “positive regulation of cell adhesion,” “leukocyte cell−cell adhesion,” “leukocyte migration,” and “regulation of T cell activation.” These findings suggest that the DEGs are mainly involved in immune responses and cell adhesion, indirectly reflecting the active tumor immune microenvironment (TME) in PTC tissues. This observation is consistent with the findings reported in the original publications of the GSE184362 dataset (PMID: 34663816; PMID: 36523593; PMID: 38609408), which also highlighted the abundance of immune cell types such as T cells, B cells, macrophages, and dendritic cells in the samples.

We fully acknowledge and appreciate your concerns and suggestions. In our ongoing research, we have collected 10 PTC samples along with matched adjacent normal tissues for single-cell RNA sequencing. We aim to investigate lysosome-associated features in PTC using both spatial transcriptomics and the latest single-cell data. Additionally, we intend to construct a reference gene set specific to PTC to achieve more accurate cell-type annotations.

Once again, thank you for your invaluable feedback and support in improving our manuscript.

Sincerely,
