# Supplementary material for: Multi-omics analysis and single-cell sequencing revealed the lysosome associated molecular subtypes and prognostic model development of papillary thyroid carcinoma
Source: PLoS One. 2025 Jun 10;20(6):e0325486. doi: 10.1371/journal.pone.0325486 (PMC12151391; doi:10.1371/journal.pone.0325486)
Supplement: Attachment — Submitted filename: Response_to_Reviewers_auresp_3.docx [file pone.0325486.s008.docx]

Dear Editor and Reviewers,

We sincerely appreciate your time and effort in reviewing our manuscript. Your insightful comments and valuable suggestions have been extremely helpful in improving the quality of our work. We have carefully addressed all the feedback and made the necessary revisions accordingly. Below, we provide detailed responses to each comment. We hope that our revisions meet your expectations, and we are grateful for any further suggestions you may have.

Reviewer #3: The authors addressed all the points raised in previous reviews and have put more thought into the comments made regarding the single cell RNA-seq portion of their manuscript.
While the authors are right that there are no standard annotations of tumor profile in the common single cell reference databases, it is not impossible to figure that out. The authors could have performed a CNV analysis on scRNAseq data using inferCNV or other R packages. Using CNV profiles the authors could tell that normal cells are the ones with no CNV and the tumor cells are the ones with.
I am not convinced that this is the maximum that can be done with the data. I advised the authors to discuss this with single cell transcriptomics experts. I am sure that if they did then that person would have told them that they need to run a copy number analysis to identify the tumor cells.
I am not satisfied with the justifications, but it seems that the authors do not want to spend more time on this. If this is the case, then the authors will have to incorporate all the justifications and explanations made to me in the manuscript in the discussion section to the readers are aware of the limitations of the single cell section. I will leave it to the editor to make a decision on this manuscript

Reply：

We sincerely thank you for your thoughtful re-evaluation of our manuscript and especially for your insightful suggestions regarding the single-cell RNA sequencing analysis.

Regarding your recommendation to perform CNV analysis using tools such as inferCNV or other R packages to identify malignant cells within the scRNA-seq dataset, we fully agree with your assessment of its value and relevance. We appreciate your emphasis on the importance of distinguishing malignant from non-malignant cells to improve the accuracy and biological interpretability of our findings.

After receiving your comments, we engaged in discussions with several researchers experienced in single-cell transcriptomics in an effort to explore the feasibility of incorporating CNV inference into our current analysis pipeline. Unfortunately, due to the lack of well-defined normal reference cells in our dataset and other technical limitations specific to our samples, none of the consulted experts could offer a clear or feasible path forward for applying CNV analysis in this context. Combined with constraints in data quality and available resources, we were unable to integrate this analysis into the current version of the study, and we sincerely apologize for this limitation. Moreover, we would like to clarify that our primary objective in conducting the scRNA-seq analysis was to characterize the broad composition of cell types within the tumor microenvironment and to explore their functional states, rather than to precisely distinguish malignant from non-malignant populations. While we acknowledge that CNV analysis would have enhanced the resolution of tumor cell identification, it was not the central focus of this study.

We have taken your advice seriously and have revised the discussion section accordingly. We now explicitly acknowledge the absence of CNV-based tumor cell identification as a limitation of our study, clarify the rationale behind this methodological decision, and emphasize the need for future research that incorporates CNV analysis to further strengthen the interpretability of single-cell data in PTC.

We are truly grateful for your constructive feedback and your continued support of our work. We hope that the additional clarifications and revisions adequately reflect our appreciation of your suggestions and our commitment to improving the rigor of our research.

**Line 521-538**
